# The Impact of Multiple Functional Layers in the Structure of Magnetic Nanoparticles and Their Influence on Albumin Interaction

**DOI:** 10.3390/ijms221910477

**Published:** 2021-09-28

**Authors:** Joana C. Pieretti, Jordan Beurton, Julián Munevar, Luiz C. C. M. Nagamine, Alain Le Faou, Amedea B. Seabra, Igor Clarot, Ariane Boudier

**Affiliations:** 1 Center for Natural and Human Sciences (CCNH), Federal University of ABC (UFABC), Santo André 09210-580, Brazil; joana.pieretti@ufabc.edu.br (J.C.P.); julianmunevar@gmail.com (J.M.); amedea.seabra@ufabc.edu.br (A.B.S.); 2 Université de Lorraine, CITHEFOR, F-54000 Nancy, France; jordan.beurton@univ-lorraine.fr (J.B.); alain.lefaou@univ-lorraine.fr (A.L.F.); igor.clarot@univ-lorraine.fr (I.C.); 3 Instituto de Física, Universidade de São Paulo, São Paulo 05508-090, Brazil; nagamine@if.usp.br

**Keywords:** magnetic nanoparticles, silver nanoparticles, Mössbauer spectroscopy, protein corona, hybrid nanoparticles

## Abstract

In nanomedicine, hybrid nanomaterials stand out for providing new insights in both the diagnosis and treatment of several diseases. Once administered, engineered nanoparticles (NPs) interact with biological molecules, and the nature of this interaction might directly interfere with the biological fate and action of the NPs. In this work, we synthesized a hybrid magnetic nanostructure, with antibacterial and antitumoral potential applications, composed of a magnetite core covered by silver NPs, and coated with a modified chitosan polymer. As magnetite NPs readily oxidize to maghemite, we investigated the structural properties of the NPs after addition of the two successive layers using Mössbauer spectroscopy. Then, the structural characteristics of the NPs were correlated to their interaction with albumin, the major blood protein, to evidence the consequences of its binding on NP properties and protein retention. Thermodynamic parameters of the NPs–albumin interaction were determined. We observed that the more stable NPs (coated with modified chitosan) present a lower affinity for albumin in comparison to pure magnetite and magnetite/silver hybrid NPs. Surface properties were key players at the NP–biological interface. To the best of our knowledge, this is the first study that demonstrates a correlation between the structural properties of complex hybrid NPs and their interaction with albumin.

## 1. Introduction

In recent years, a remarkable advance has been achieved in the field of nanoparticles (NPs) designed for biomedical applications [1]. Hybrid nanomaterials stand out due to the possibility of combining multiple functions in a single structure, providing new opportunities in both the diagnosis and treatment of several diseases (i.e., cancer and infectious diseases) [2,3], or as an innovative theragnostic approach [4]. When compared to bulk materials, NPs present improved interactions with biomolecules due to their intrinsic properties and small size, generating an NP–biological interface. At the surface of the particles, thermodynamic exchanges occur in a dynamic interaction with biomolecules, such as nucleic acids, enzymes, and proteins [1,5]. Thus, NP size, as well as the intrinsic physicochemical properties have to be put forth, as they are influenced by the combination of each component of the hybrid structure [6].

Magnetite-based NPs (Fe_3_O_4_ NPs) are usually designed for intravenous applications, which permit localizing them at the chosen target using an external magnetic field [7]. However, after injection, these hybrid nanomaterials will immediately interact with the biological molecules or cells, which may lead to positive or negative effects according to the nanomaterial biodistribution and bioactivity [8,9]. It is well-established that this interaction is influenced by the NP surface charge, hydrophobicity, size, shape, composition, and the binding affinity of biomolecules for its surface [10]. The influence of the NPs surface modification and their biological effects have already been reported: (i) pegylated NPs demonstrate a prolonged blood circulation and low targetability; (ii) NPs modified with specific ligands have limited blood circulation but accumulation in the targeted organ; (iii) NPs coated with a combination of shield molecules enable a prolonged blood circulation and a high targetability [11].

Considering the increasing progress regarding the engineering of hybrid nanomaterials, combining several NPs in one unique nanostructure induces surface modification, which is also obtained by adding polymers and/or functional, and/or drug-releasing coatings [3,12]. Our group previously demonstrated the antibacterial and antitumoral properties of a hybrid nanostructure based on Fe_3_O_4_, and silver NPs (AgNPs), coated with a modified chitosan polymer, thiolated chitosan (TCS) [3], which offers a platform for further chemical modifications [13,14]. The goal was to combine the magnetic properties of Fe_3_O_4_ NPs with the antimicrobial and the antitumoral effects of AgNPs [15,16]. In this scenario, structural properties of the Fe_3_O_4_ magnetic core must be evaluated, as these NPs tend to rapidly oxidize from magnetite to maghemite, especially considering the addition of multiple layers on its surface [17]. 

The fate of the NPs is directly related to their interaction with biomolecules after an in vivo administration, in which the protein corona plays a main role [18]. The formation of protein corona directly affects the NPs targetability and efficiency, and it may also promote side effects such as blood coagulation, cardiovascular diseases, or pro-inflammatory effects [18]. Albumin is an important blood protein that presents in major concentration in animals’ plasma; therefore, it is usually used as a model protein for drug interaction studies [19]. Moreover, albumin is also employed as a drug nanocarrier, mainly for anticancer drugs. Thus, nab-paclitaxel (Abraxane) was the first albumin-stabilized nanoformulation developed for breast cancer treatment to be approved by the Food and Drug Administration (FDA) [20]. Still, the mechanisms of protein corona formation are still poorly understood, mainly for complex NPs. As discussed in the present report. Fe_3_O_4_ NPs have demonstrated great prospects in the biomedical field, and studies focusing on Fe_3_O_4_ NPs interactions with albumin have already been reported [21,22]. Fe_3_O_4_ NPs spontaneously interacted with albumin and other proteins, depending on the NPs and proteins’ properties. Thus, we aimed to investigate the influence of the addition of multiple functional layers on the surface of Fe_3_O_4_ NPs and their interaction with albumin.

**Figure 1:**
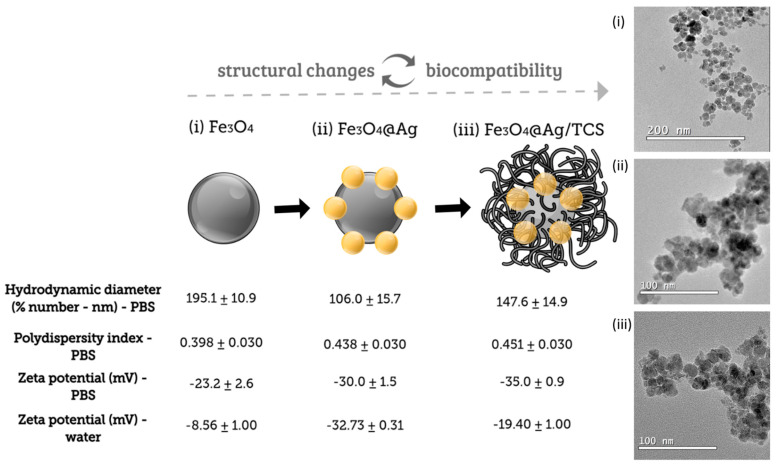
Schematic representation of the NPs evaluated in this work, and values of hydrodynamic diameter expressed by percentage of number; PDI (in PBS medium) and zeta potential values for Fe_3_O_4_ NPs, Fe_3_O_4_@Ag NPs, and Fe_3_O_4_@Ag/TCS NPs in water and in PBS. TEM micrographs of (i) Fe_3_O_4_ NPs, (ii) Fe_3_O_4_@Ag NPs, and (iii) Fe_3_O_4_@Ag/TCS NPs showing, respectively, average solid-state diameters of 14.4 nm ± 5.0, 13.6 nm ± 2.5, and 14.6 nm ± 1.5.

Herein, we have studied the interactions between Fe_3_O_4_-based NPs and albumin. The three studied NPs (Figure 1) were obtained after two successive layer additions on the magnetic core, as follows: (i) pure Fe_3_O_4_ NPs, without coating; (ii) Fe_3_O_4_ NPs coated with Ag NPs, leading to Fe_3_O_4_@Ag NPs; (iii) Fe_3_O_4_@Ag NPs coated with TCS (Fe_3_O_4_@Ag/TCS). The complex structure of these NPs directly impacted the morphological and structural parameters as well as their surface chemistry. Therefore, the polymorphism of Fe_3_O_4_ was investigated using Mössbauer spectroscopy, which is considered as a refined technique to investigate the local Fe environment [23]. The surface structure of the hybrid NPs may correspond to different affinity levels for albumin comparing the different structures, resulting in differences in thermodynamic parameters (∆S, ∆H, and ∆G) and result in the binding forces between the NPs and the proteins [24]. Thus, we studied the binding properties of each nanostructure represented in Figure 1 to albumin, in order to comprehend the impact of multilayered NPs in the formation of protein corona. 

## 2. Results and Discussion

### 2.1. Morphological Characterization of Fe_3_O_4_ NPs, Fe_3_O_4_@Ag NPs, and Fe_3_O_4_@Ag/TCS NPs

The syntheses of Fe_3_O_4_ NPs, Fe_3_O_4_@Ag NPs, and Fe_3_O_4_@Ag/TCS NPs were successfully performed through the proposed route, employing green tea extract as a non-toxic reducing agent of silver ions (Ag^+^) to metallic silver (Ag NPs) onto the surface of the Fe_3_O_4_ magnetic core, as previously described [3]. We have previously reported that the hybrid NPs presented a mass proportion of 87% of Fe_3_O_4_ NPs and 13% of Ag NPs in one stable Fe_3_O_4_@Ag NPs nanostructure [3]. The surface of the hybrid nanostructure Fe_3_O_4_@Ag NPs was functionalized with TCS, which can be further functionalized. Our previous publication reported the morphological and physicochemical characterizations of the NPs by different techniques [25]. Considering that both size and surface properties directly interfere with the functionality of NPs [26,27], the hydrodynamic diameters, polydispersity index values, and zeta potentials were measured to determine the influence of the surface of the three NPs on their properties. The results are presented in Figure 1.

Fe_3_O_4_ NPs presented the highest hydrodynamic diameter value, which decreased after AgNPs coating. The observed pattern was expected, as bare magnetic Fe_3_O_4_ NPs tend to agglomerate because the NPs are not stabilized with a polymeric or metallic coating [28]. The coating of Fe_3_O_4_@Ag NPs with TCS slightly increased the diameter of Fe_3_O_4_@Ag/TCS NPs because of the presence of a polymeric layer. However, Fe_3_O_4_ NPs showed the highest hydrodynamic size, since uncoated NPs tend to aggregate in aqueous medium due to surface interactions between these NPs. In PBS medium, all NPs presented a moderate polydispersity index, and the colloidal suspension demonstrated negative partial charges. In water, the zeta potential values better represent the surface charge of each NP in a medium containing less ions, compared with PBS medium. Zeta potential values indicated that Fe_3_O_4_ NPs showed a lower negative value of surface charge when compared to the other NPs (i.e., Fe_3_O_4_@Ag NPs and Fe_3_O_4_@Ag/TCS NPs), which corroborates with a higher aggregation tendency [28], while a high negative surface charge was observed for Fe_3_O_4_@Ag NPs, which is characteristic of NPs reduced with green tea extract, as phytochemicals such as polyphenols remain attached on the surface of the nanomaterial [15]. Finally, the coating with TCS slightly increased the zeta potential due to the positive surface charge of the polymer [14]. The dynamic light scattering characterizations indicated different sizes and surface properties for each NPs, reinforcing the need in the evaluation of NP–protein interaction to better understand the influence of each layer on the biological fate and function of these NPs [26]. Multilayered NPs directly influence the properties of the final material. 

### 2.2. Evaluation of the Fe_3_O_4_ Polymorphs in the NP

Conventional techniques to investigate the local Fe environment in iron-based NPs are not completely efficient, which is mostly due to the similarity in the iron oxides structures [29]. Thus, analyses such as X-ray diffraction, Fourier transform infrared spectroscopy (FTIR), and even X-ray photoelectron spectroscopy (XPS) cannot precisely characterize iron oxide structures. Considering that Fe_3_O_4_ NPs under ambient conditions spontaneously oxidize into maghemite (γ-Fe_2_O_3_ NPs) [30], Mössbauer spectroscopy appears as the adequate technique to monitor not only the interconversion between the iron oxides phase but also the NP structure after the addition of each layer. This is possible due to its unique sensitivity to distinguish the different Fe oxidation states, local site symmetries, and magnetic interactions present at the Fe site. Moreover, spectra recorded below the characteristic NP blocking temperature allow in some cases the clear identification of magnetite/maghemite mixtures. Using simple models, it is possible to obtain important information about ratios between the surface and core in NPs. Thus, we investigated the structure and composition of the NPs (Fe_3_O_4_ NPs, Fe_3_O_4_@Ag NPs, and Fe_3_O_4_@Ag/TCS NPs) using low-temperature Mössbauer Spectroscopy. 

**Figure 2:**
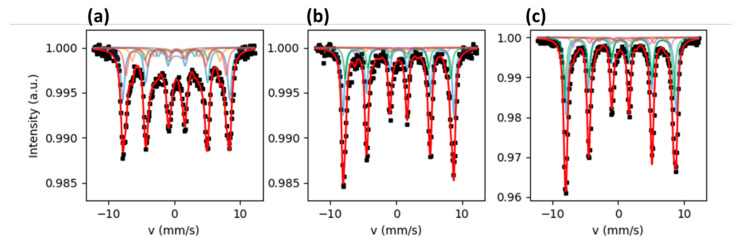
Mössbauer spectra of (**a**) Fe_3_O_4_ NPs, (**b**) Fe_3_O_4_@Ag NPs, and (**c**) Fe_3_O_4_@Ag/TCS NPs obtained at 80 K. The solid lines represent the least square fits to the Mössbauer spectra for each site and the total spectrum.

Mössbauer spectra of Fe_3_O_4_, Fe_3_O_4_@Ag, and Fe_3_O_4_@Ag/TCS NPs were obtained at 80 K, displaying six broad resonance lines, as expected for blocked SPIONs (superparamagnetic iron oxide NPs), as shown in Figure 2. The corresponding spectra were fitted using six Fe sites, and the average isomer shift and the spectral areas were used to estimate the percentage of Fe_3_O_4_ in the NP. The choice criteria of the Fe sites aimed to obtain the best fit.

By comparing our recorded spectra to those reported for pure nano-magnetite [31], it became evident that the presence of the octahedral Fe^2+^ subspectra in our spectra is not clear, resembling more the spectrum obtained with maghemite NPs [32]. For this reason, the analysis of the Mössbauer spectra was performed using a novel approach based on the determination of the isomer shift « center of gravity » [33], from where it is possible to determine the ratio of γ-Fe_2_O_3_ and Fe_3_O_4_ admixtures, independent of their morphology or microstructure. This analysis is independent of the chosen model; the only requirement is to obtain a fit with a low χ^2^ coefficient.

We fitted our spectra using six sextets, keeping the ratio between the resonance lines as 3:2:1. Depending on the overall resonance line widths, the sites were grouped by constraining the isomer shift and allowing different values of magnetic hyperfine field B_HF_. The larger the line width, the larger the number of sites per isomer shift value. The spectra and their fits are shown in Figure 2, and the corresponding hyperfine parameters obtained from the fits are reported in Table 1.

We obtained the corresponding values of the ratio between the number of Fe atoms present in the form of Fe_3_O_4_ upon the total number of Fe atoms in the NPs, defined as α, by using the average isomer shift δ, which is computed from the isomer shifts obtained from the fit and weighted by the spectral areas of each site used. Equation (1) was used [33]:

(1) $$\alpha =\frac{\delta^{-}\left(80\;K\right)-0.93a_{\gamma }-0.07\delta_{0}}{0.93\left(a_{\mathrm{Fe}}-a_{\gamma }\right)+0.07\;m}$$

where m = 0.2135(7) mm/s, δ_0_ = 0.3206(2) mm/s, a_γ_ = 0.4350(2) mm/s, and a_Fe_ = 0.6470(2) mm/s [33]. The corresponding values for α are 0.12 ± 0.05, 0.20 ± 0.05, and 0.16 ± 0.05, respectively for the Fe_3_O_4_, Fe_3_O_4_@Ag, and Fe_3_O_4_@Ag/TCS.

The α values can be explained assuming a core–shell model, where we associate this Fe_3_O_4_ to the Fe oxide in the core of the NP and γ-Fe_2_O_3_ forming in its shell. The greater ratios of Fe_3_O_4_ observed for the Ag-coated NPs suggest that the oxidation from magnetite to maghemite is prevented by this addition. Furthermore, despite the generic model used to fit the spectra, it is possible to observe the possible influence of the NP size and its related relaxation effect. The mean blocking temperature for these NPs is above 80 K, which ensures the observation of the resonance line splitting due to the magnetic hyperfine field at the Fe nucleus. However, by considering sites with B_HF_ as low as 8 T, one can think that even at this temperature, there may be NPs close to their superparamagnetic state, therefore suggesting a somewhat large distribution of NP sizes. This result can be directly correlated to previous DLS results, in which all NPs have demonstrated a moderate polydispersity index, suggesting large size distribution.

### 2.3. Interaction between NPs and Albumin

#### 2.3.1. Influence of Albumin Adsorption on the Surface Properties of NPs

**Figure 3:**
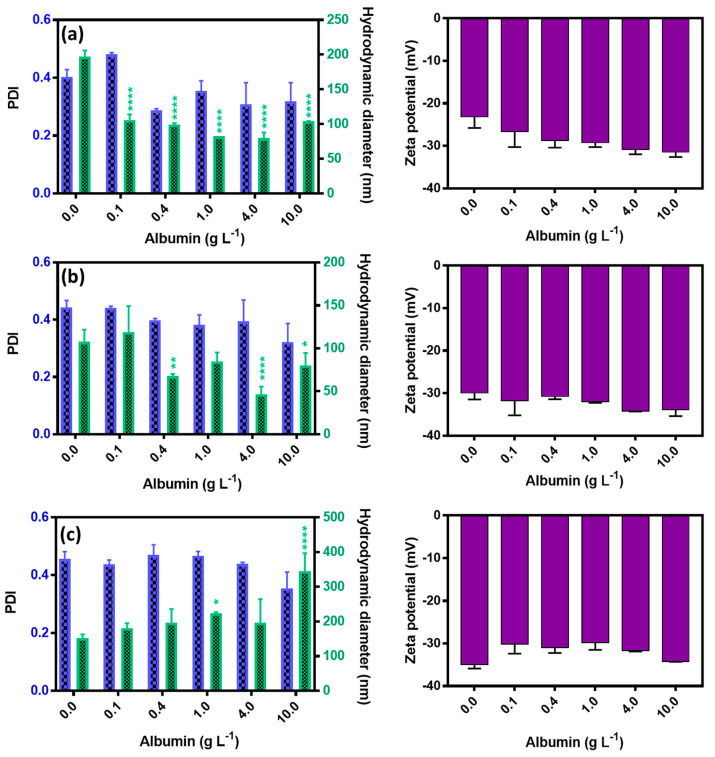
Influence of increased concentrations of albumin on the physicochemical data of (**a**) Fe_3_O_4_ NPs, (**b**) Fe_3_O_4_@Ag NPs, and (**c**) Fe_3_O_4_@Ag/TCS NPs. Results show the values of hydrodynamic diameter (nm), measured in percentage of number, polydispersity index (PDI), and zeta potential (mV). * *p* < 0.0360, ** *p* < 0.0024, and **** *p* < 0.0001, calculated using two-way ANOVA.

The three synthesized NPs differ by their structure and hence their surface properties. NPs were put in contact with increasing concentrations of albumin. The values of hydrodynamic diameter, polydispersity index (PDI), and zeta potential for Fe_3_O_4_, Fe_3_O_4_@Ag, and Fe_3_O_4_@Ag/TCS NPs after incubation are shown in Figure 3.

Non-polymer-coated NPs (Fe_3_O_4_ and Fe_3_O_4_@Ag NPs) presented a decrease of the hydrodynamic diameter with an increasing concentration of albumin. On the opposite, Fe_3_O_4_@Ag/TCS NPs presented a significant progressive increase. Such an observation is often described [24,34], as it is expected that the hydrodynamic diameter of both inorganic and organic NPs increases due to the deposition of albumin, adding an extra layer on the surface [24,34], although an opposite effect has also been observed [23]. The size reduction for both Fe_3_O_4_ and Fe_3_O_4_@Ag NPs might reflect a steric stabilization proportioned by the albumin coating. Recently, albumin coating of NPs has been used for both stabilizing and providing a sustained circulation of NPs [35,36], corresponding to the stabilizing effect observed for the non-coated NPs described in this work. For Fe_3_O_4_@Ag/TCS NPs, already presenting a polymeric coating of TCS, the size increase might be related to binding forces between the protein and the polymer, forming an extra albumin layer on the NP surface [24,34]. No statistical difference was observed for the polydispersity index of the three evaluated NPs in the presence or absence of albumin. Moreover, the zeta potential values agreed with the expected ones. Fe_3_O_4_, Fe_3_O_4_@Ag, and Fe_3_O_4_@Ag/TCS NPs already presented negatively charged surfaces, and albumin, which globally bears negative charges at pH 7.4 [23], did not modify the results.

#### 2.3.2. Determination of the Type of Complex between NPs and Albumin 

Many studies using different approaches have been performed in order to shed light on the interaction between NPs and proteins, such as the consequence of protein binding on the size and stability of NPs. However, results are intrinsically dependent on the experimental conditions, since the separation of the NPs from the unbound albumin may impact the complex protein–NP by equilibrium destabilization [8]. A different possibility is the direct investigation of binding forces between the NPs and proteins. In this regard, the fluorescence quenching is an ideal technique, as it is based on the modification of intrinsic tryptophan fluorescence through a non-destructive method [19]. The fluorophore interaction with drugs [37] or NPs [38] induces a modification of the amino acid environment, which leads to significant modifications, i.e., quenching. 

**Figure 4:**
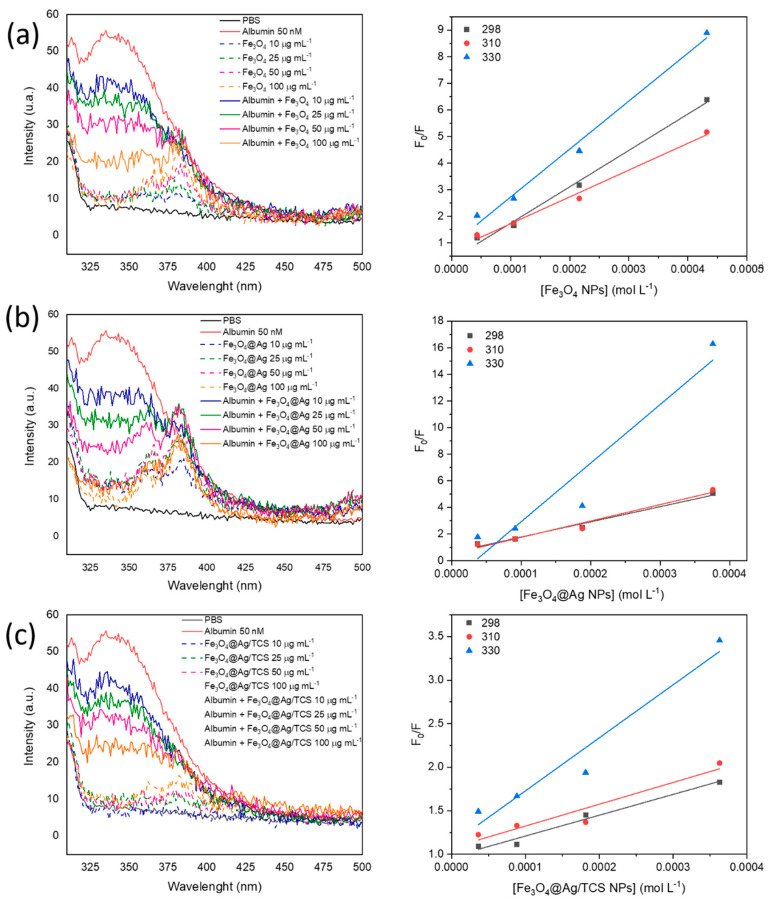
Fluorescence spectra and Stern-Volmer plot of F_0_/F vs. NPs concentration ([NP]) for (**a**) Fe_3_O_4_ NP, (**b**) Fe_3_O_4_@Ag, and (**c**) Fe_3_O_4_@Ag/TCS incubated with albumin at 298, 310, and 330 K. The concentrations of NPs were given in mol L^−1^, taking into account the molar mass of magnetite NPs and the proportion of this NP in the other materials (87% in Fe_3_O_4_@Ag and 84% in Fe_3_O_4_@Ag/TCS).

A decrease of albumin intrinsic fluorescence was observed, which is related to the concentrations of Fe_3_O_4_, Fe_3_O_4_@Ag, and Fe_3_O_4_@Ag/TCS NPs (Figure 4). NPs without albumin presented a negligible fluorescence. The initial experimental step was the determination of the nature of the complex between NP and albumin, as their interaction may be either static or dynamic [19]. The type of interactions was determined using Stern-Volmer equation, which resulted in the graphics shown in Figure 4 and data presented in Table 2. 

As observed in Table 2, the values of K_q_, were higher than 2 × 10^10^ L mol^−1^ s^−1^ (maximum scattering collision quenching constant) for all NPs, indicating that the formed complex was stable and the distance between the tryptophan residue and the NPs remained constant [37]. Furthermore, the values of K_q_ increased with the temperature for all three NPs evaluated, as observed for a magnetic nanofluid by Paul et al. [39]. Therefore, the equilibrium of complex formation between NP and albumin is written as Equation (2):

(2) $$\mathrm{NP}+\;\mathrm{Protein}\;\overset{\mathrm{Ka}}{\to }\;[\mathrm{NP},\;\mathrm{Protein}]$$

where Ka is the affinity constant.

#### 2.3.3. Determination of Interaction Affinity between NPs and Albumin

**Figure 5:**
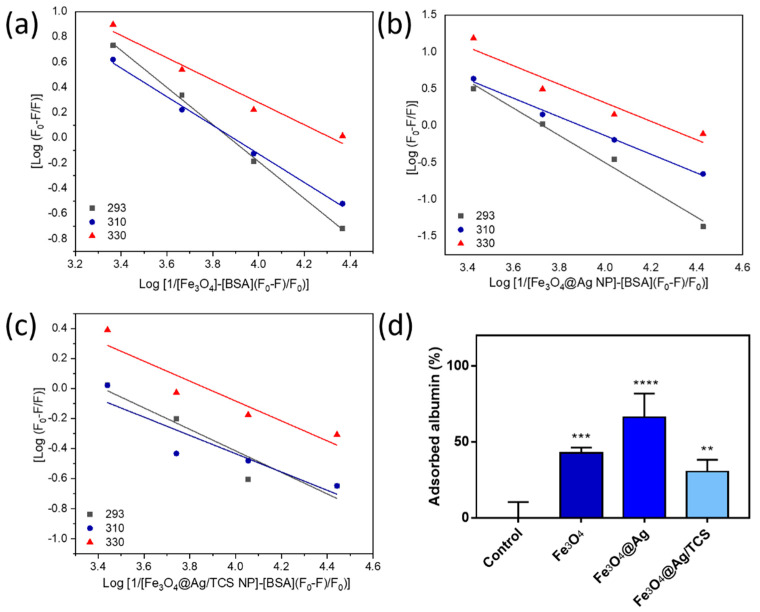
Plot of Log ((F_0_ − F)/F) vs. Log (1/([NP] − [BSA](F_0_ − F)/F_0_)) for (**a**) Fe_3_O_4_ NPs and (**b**) Fe_3_O_4_@Ag NPs, and (**c**) Fe_3_O_4_@Ag/TCS NPs incubated with albumin, as a function of the temperature; (**d**) adsorbed albumin (%) after the incubation of 0.4 g L^−1^ of protein with 200 µg mL^−1^ of Fe_3_O_4_ NPs, Fe_3_O_4_@Ag NPs, and Fe_3_O_4_@Ag/TCS NPs. ** *p* < 0.0013, *** < 0.0006, and **** *p* < 0.0001, comparing to control, using Tukey’s multiple comparisons test.

Then, the obtained results were further analyzed in order to determine the thermodynamic parameters. From Equation (2) and the van’t Hoff plot, thermodynamic constants were obtained for Fe_3_O_4_ NPs, Fe_3_O_4_@Ag NPs, and Fe_3_O_4_@Ag/TCS NPs and are shown in Figure 5. Data obtained from Figure 5 were further analyzed, and the results are summarized in Table 3.

Of the three NPs, Fe_3_O_4_@Ag/TCS NPs presented the lowest affinity for albumin, as evidenced by a lower affinity constant (Ka) at all the tested temperatures. On the contrary, Fe_3_O_4_ NPs and Fe_3_O_4_@Ag NPs exhibited higher and close Ka values, indicating quite similar affinities. In addition, the quantification of unabsorbed protein in the supernatant after the incubation confirms these tendencies (Figure 5d).

#### 2.3.4. Determination of Type of Interaction between NPs and Albumin

In addition to the affinity, the values of the thermodynamic parameters (ΔS and ΔH and ΔG) were determined (Table 3). The interaction of the NPs with albumin was spontaneous: ΔG values were negative. According to Ross and Subramanian [22], the positive values of the thermodynamic constants (ΔS and ΔH) for Fe_3_O_4_, Fe_3_O_4_@Ag, and Fe_3_O_4_@Ag/TCS NPs evidenced hydrophobic interactions with albumin [40]. The NP–protein association is frequently driven by weak interactions, and its mechanism enables the surface stabilization of each particle [41]. Hydrophobic interaction is the result of a partial withdrawal of the nonpolar group of water molecules and the solvation layer of the three NPs and/or albumin.

Understanding the interaction between proteins and NPs is fundamental for predicting therapeutic responses. In this work, we investigated the interaction mechanisms between albumin and NPs that led to the formation of a protein layer. The structural changes of the protein chain after interaction with NPs may positively or negatively affect the in vivo fate of NPs in terms of toxicity and cellular uptake [38]. For example, Minchin et al. demonstrated that the interaction of silica NPs with albumin changed the structure of the latter, leading to the exposure of a hidden epitope, as recognized by macrophages [42]. In contrast, after its adsorption on Fe_3_O_4_ NPs, transferrin undergoes structural changes, losing its iron carrier activity [43]. In this work, the most stabilized NPs (i.e., Fe_3_O_4_@Ag/TCS NPs) presented the minimal albumin adsorption, but this phenomenon could not be completely abolished as already observed for other NPs in the literature [24].

## 3. Materials and Methods

### 3.1. Chemicals

Bovine serum albumin (BSA, the absence of oligomerization was previously checked using dynamic light scattering and size exclusion chromatography), chitosan (75% deacetylation, medium molecular weight), ethanol (99%), iron chloride II tetrahydrate (FeCl_2_∙4H_2_O), *N*-(3-dimethylaminopropyl)-*N*′-ethylcarbodiimide hydrochloride (EDC), phosphate saline buffer (PBS) pH 7.4 composed of 3.2 mmol L^−1^ KH_2_PO_4_, 7.0 mmol L^−1^ K_2_HPO_4_, 149.9 mmol L^−1^ NaCl, and NaOH solution for pH adjustment, thioglycolic acid (TGA) were purchased from Sigma-Aldrich, MO, USA. Acetic acid (HAc), acetone, ammonium hydroxide (NH_4_OH), iron chloride III hexahydrate (FeCl_3_∙6H_2_O), hydrochloric acid (HCl), silver nitrate (AgNO_3_), and sodium hydroxide (NaOH) were obtained from Synth, Diadema, SP, Brazil. Powdered green tea (*Camellia sinensis*) was obtained from Sumioka Shokuhin Kabushikikaisha, Hiraguti, Japan. Sodium chloride was purchased from VWR chemicals, Fontenay-sous-Bois, France. All preparations and experiments were performed using fresh Milli-Q water. 

### 3.2. Synthesis of Fe_3_O_4_, Fe_3_O_4_@Ag NPs, Fe_3_O_4_@Ag/TCS NPs 

Hybrid Fe_3_O_4_@Ag NPs were synthesized as previously reported [3]. Firstly, Fe_3_O_4_ NPs were synthesized through the chemical co-precipitation method of Fe II and Fe III ions in the presence of NH_4_OH. The precipitate was washed several times with water and ethanol and separated with a magnet. Fe_3_O_4_ NPs were freeze-dried and stored in the dark before use. To obtain Fe_3_O_4_@Ag NPs, Fe_3_O_4_ NPs were resuspended in deionized water and homogenized with AgNO_3_ solution (0.65 mol L^−1^) for 1 h, protected from light. A volume of 60 mL of green tea extract was added to the previous suspension, the pH of the final suspension was adjusted to 11.0 using NaOH (1.0 mol L^−1^), and the nanomaterial was stirred for 2 h. Fe_3_O_4_@Ag NPs were washed with water, freeze-dried, and stored in the dark.

Fe_3_O_4_@Ag NPs were coated with thiolated chitosan (TCS). TCS was synthesized through the reaction of commercial CS with TGA in the presence of a carbodiimide catalyst (EDC) [14]. Fe_3_O_4_@Ag NPs were resuspended in water, and 20% m/v of TCS was added. The final mixture was stirred for 2 h, washed with water, freeze-dried, and stored in the dark. 

### 3.3. Characterization of Fe_3_O_4_, Fe_3_O_4_@Ag NPs, and Fe_3_O_4_@Ag/TCS NPs

#### 3.3.1. Morphological Characterizations

The hydrodynamic diameter and the polydispersity index of Fe_3_O_4_, Fe_3_O_4_@Ag NPs, and Fe_3_O_4_@Ag/TCS NPs were determined by dynamic light scattering (DLS), using a Zetasizer nanoseries (Nano-ZS) coupled with a 633 nm laser and adjusted with a backscattered angle of 173° (Malvern Instruments, UK). The surface zeta potential was evaluated by laser Doppler velocimetry, under the same conditions as DLS measurements. All measurements were performed at 25 °C, with 120 s thermal equilibration, in plastic cuvettes and a disposable folded capillary zeta cell (10 mm path length). All NPs were resuspended in PBS pH 7.4 for 1 h in an ultrasound bath at 32 kHz (Prolabo, Germany). Zeta potential was also measured in water to exclude ionic charge from PBS. Results are expressed as mean ± standard deviation from 3 independent experiments. TEM images of NPs were acquired using a JEM 2100 B6 transmission electron microscope (JEOL, Akishima, Tokyo), with resolution of 0.25 nm point-to-point and acceleration voltage of 200 kV. Samples were dispersed in water and drop casted onto copper grids for analysis.

#### 3.3.2. Mössbauer Spectroscopy

The local structural and magnetic properties of the iron oxide phases were investigated by ^57^Fe Mössbauer Spectroscopy, specifically to obtain the composition of Fe_3_O_4_, Fe_3_O_4_@Ag, and Fe_3_O_4_@Ag/TCS NPs. Energy-dependent gamma ray transmission spectra were obtained at 80 K (Cryostat JANIS, Model SVT-490) from powdered samples of the above-described NPs mounted on polymer sample holders. A ^57^Co:Rh source was used to obtain the 14.4 keV γ rays detected with a proportional gas detector (High Voltage Supply and amplifier, Ortec 556 and 572A); a standard transmission spectrometer moving the source with a constant velocity transducer was employed (Model MR-354), keeping the source at room temperature.

### 3.4. Interaction between NPs and Albumin

#### 3.4.1. Influence of Albumin Adsorption on Fe_3_O_4_, Fe_3_O_4_@Ag NPs, and Fe_3_O_4_@Ag/TCS

For the evaluation of the influence of albumin in the hydrodynamic size of Fe_3_O_4_ NPs, Fe_3_O_4_@Ag NPs and Fe_3_O_4_@Ag/TCS NPs were individually dispersed in a Transsonic ultrasound bath at 32 kHz (Prolabo, Germany) for 1 h and then were diluted to a final concentration of 200 µg mL^−1^. Furthermore, NP suspensions were incubated with albumin at final concentrations of 0.4, 1.0, 4.0, and 10.0 g L^−1^, and prepared in PBS pH 7.4 at 37 °C for 4 h. The NPs were separated from the supernatant using a magnet and resuspended in the same volume of deionized water. The fluorescence of the supernatant due to unbound albumin was measured using a spectrofluorometer (FP-8300iRM, Jasco, France), using a quartz cuvette with a light path of 10 × 4 mm (Hellma Analytics, Germany) at 280 ± 5 nm excitation length and 345 ± 5 nm emission length, at low sensibility. The percentage of albumin attached to the NPs was calculated based on a calibration curve of albumin ranging from 0.1 to 10.0 g L^−1^. Resuspended NPs were further characterized by dynamic light scattering (DLS), in order to measure the hydrodynamic size, polydispersity index, and zeta potential, as described above. DLS results were compared to pure NPs dispersed in PBS. The statistical significance was determined using one-way ANOVA and Tukey’s test. Differences were considered significant for *p* < 0.05.

#### 3.4.2. Spectrofluorometric Analyses of the Interaction between NPs and Albumin

To analyze the interaction between NPs and albumin, fluorescence quenching experiments were performed in a spectrofluorometer [23]. The parameters were set as previously described. The concentration of albumin was kept constant (50 nmol L^−1^, in PBS), as this concentration resulted in an ideal fluorescence intensity for the experiments, and the concentration of Fe_3_O_4_ NPs, Fe_3_O_4_@Ag NPs, and Fe_3_O_4_@Ag/TCS NPs varied from 10 to 100 µg mL^−1^ (43 to 432 μmol L^−1^ of Fe_3_O_4_ NPs in each nanoparticle). Samples were incubated for 20 min at 3 different temperatures: 298 K, 310 K, and 330 K. Blank samples (NPs without albumin) were tested to ensure there was no significant signal from the matrix.

Mathematical equations were applied to model quenching mechanism. Firstly, the Stern-Volmer equation (Equation (3)) was applied to determine the type of complex resulting from the interaction of albumin with each type of NP [24].

(3) $$\frac{F_{0}}{F}=1+K_{q}\tau_{0}\left[\mathrm{NP}\right]=1+K_{\mathrm{sv}}\left[\mathrm{NP}\right]$$

in which F_0_ is the fluorescence intensity of albumin (50 nmol L^−1^) without NPs; F is the fluorescence of albumin in the presence of Fe_3_O_4_ NPs, Fe_3_O_4_@Ag NPs, or Fe_3_O_4_@Ag/TCS NPs; τ_0_ is the lifetime of albumin without quencher (10^−8^ s) [24]. K_q_ is the quenching constant and K_sv_ is the Stern–Volmer quenching constant; [NP] corresponds to the molar concentration of Fe_3_O_4_ NPs, Fe_3_O_4_@Ag NPs, and Fe_3_O_4_@Ag/TCS NPs, based on the molar percentage of Fe_3_O_4_ for each NP.

Afterwards, Equation (4) was used to calculate the number of binding sites (n), as well as the binding constant (Ka), in which [BSA] refers to the concentration of albumin used in this assay [37]:

(4) $$\log\left(\frac{F_{0}-F}{F}\right)=n\log\mathrm{Ka}-n\log\left(\frac{1}{\left[\mathrm{NP}\right]-\left(\;\frac{F_{0}-F\left[\mathrm{BSA}\right]}{F_{0}}\right)}\right)$$

Employing the values obtained for Ka, a van’t Hoff plot was used (lnKa = f(1/T)) and the variations of enthalpy (ΔH) and entropy (ΔS) were calculated, using Equation (5) [26].

(5) $${\mathrm{lnK}}_{A}=\frac{-\Delta H}{\mathrm{RT}}+\frac{\Delta S}{R}$$

where R is the universal gas constant (R = 8.314 J mol^−1^ K^−1^) and T is the absolute temperature. Values of Gibbs free energy (ΔG) were calculated with Ka result, using Equation (6).


(6) ΔG=−RTlnKa

Values of ΔG indicate if the interaction between albumin and each type of NP is spontaneous or not. Using the ΔH and ΔS, the binding energies between albumin and each type of NP were deduced [44].

## 4. Conclusions

In this work, we aimed to elucidate the influence of the addition of two successive layers to a Fe_3_O_4_ NP. The developed material presents promising potential in the biomedical field, especially for antibacterial and antitumoral applications [3,12,45]. The addition of each layer affects the structure of the final NP, directly impacting its properties. We observed that the addition of inorganic and organic layers changes not only the morphology/size but also the local site symmetries, the magnetic interactions, and the aggregation of the NPs. The coating with AgNPs and TCS polymer diminished the core degradation (oxidation of magnetite to maghemite) and the polydispersity of the final materials, as highlighted by Mössbauer spectroscopy. This effect directly correlates to the protein-NP interaction, in which less stabilized NPs have a higher binding to albumin. As we outlined in this article, the protein adsorption on the surface of nanoparticles is the key point that will govern their therapeutic efficacy, their bioavailability, and their residence time in the bloodstream. According to the Vroman effect [46], protein adsorption on the NP surface is a dynamic phenomenon. In blood, there is a sequential adsorption on the surface of a foreign body. Initially, the protein corona is composed of the proteins quantitatively abundant (mainly albumin), which are then progressively replaced by more specialized proteins presenting a stronger affinity for the NP. Moreover, the nature of the primary interaction with albumin is of crucial importance, since two opposite effects may result. In fact, the adsorption of albumin in the native state (i.e., without alteration of its structure) allows improvement of the furtivity of the nanoparticles, which is in favor of the residence time and a therapeutic efficacy [47]. By contrast, a significant modification of the albumin conformation on the surface of the nanoparticles would be responsible of a pro-inflammatory response and a rapid excretion [44]. Thus, albumin was chosen for these preliminary studies, and this work will be pursued mainly along two objectives. The first will study the conformation adopted by albumin on the surface of nanoparticles. Then, the second will be completed by focusing on more complex matrices such as human serum to obtain a further insight into the protein/NP interactions (kinetics, category of proteins, corona composition, etc.).

The preliminary approach detailed in this paper might be of great importance for the study of not only iron oxide-based NPs but also any multilayered NPs, which are more and more proposed for diagnostic and therapeutic purpose. 

## Figures and Tables

**Table 1:** Isomer shift, quadrupole splitting, magnetic hyperfine field, and spectral area of the Mössbauer spectrum of Fe_3_O_4_ NPs, Fe_3_O_4_@Ag NPs, and Fe_3_O_4_@Ag/TCS NPs, in which ΔEQ is the quadrupole splitting and B_HF_ is the magnetic hyperfine field.

Fe_3_O_4_ NPs				
	δ (mm/s)	ΔEQ (mm/s)	B_HF_ (T)	% Area
Site 1	0.453(7)	−0.06(2)	50.3(1)	42.0
Site 2		−0.08(4)	41.4(2)	10.9
Site 3		−0.04(7)	29.7(2)	9.9
Site 4	0.446(9)	−0.08(2)	47.2(1)	22.5
Site 5		0.13(8)	20.6(3)	7.0
Site 6		0.11(7)	8.1(2)	7.6
				
**Fe_3_O_4_@Ag NPs**				
Site 1	0.451(5)	0.00(1)	52.2(1)	59.4
Site 2		−0.11(20)	30.5(5)	3.2
Site 3	0.426(9)	−0.03(2)	49.7(1)	27.0
Site 4		0.46(36)	20.6(1.4)	1.1
Site 5	0.703(34)	0.08(8)	42.9(3)	5.7
Site 6		−0.31(14)	8.3(5)	3.5
				
**Fe_3_O_4_@Ag-TCS NPs**				
Site 1	0.480(3)	0.18(1)	51.7(2)	47.1
Site 2		0.17(6)	41.4(2)	2.6
Site 3	0.386(4)	−0.23(1)	51.2(1)	36.9
Site 4		0.54(5)	30.3(2)	3.5
Site 5	0.559(4)	0.01(3)	45.9(1)	6.2
Site 6		−0.38(5)	8.5(2)	3.6

**Table 2:** Parameters estimated for Fe_3_O_4_ NPs, Fe_3_O_4_@Ag NPs, and Fe_3_O_4_@Ag/TCS NPs fitted according to Stern-Volmer equation from Figure 4.

NP	T (K)	K_sv_ (L mol^−1^)	R^2^	K_q_ (10^10^ mol^−1^ L^−1^)
**Fe_3_O_4_**	298 310 330	1.37 × 10^4^ 1.01 × 10^4^ 1.80 × 10^4^	0.99 0.99 0.99	13.7 10.1 18.0
**Fe_3_O_4_@Ag**	298 310 330	1.14 × 10^4^ 1.23 × 10^4^ 1.59 × 10^4^	0.98 0.97 0.92	11.4 12.3 15.9
**Fe_3_O_4_@Ag/TCS**	298 310 330	2.39 × 10^3^ 2.50 × 10^3^ 6.09 × 10^3^	0.98 0.92 0.95	2.39 2.50 6.09

**Table 3:** Thermodynamic parameters of the NPs–albumin complex obtained from the graphs of Figure 5.

NP	T (K)	Ka (L mol^−1^)	∆H (kJ mol^−1^)	∆S (J mol^−1^ K^−1^)	∆G (kJ mol^−1^)	Interaction Mechanism
**Fe_3_O_4_**	298	7.45 × 10^3^	19.63	140.51	−22.09	Hydrophobic interactions
	310	7.72 × 10^3^			−23.08	
	330	2.08 × 10^4^			−27.28	
**Fe_3_O_4_@Ag**	298	5.34 × 10^3^	13.13	118.86	−21.27	Hydrophobic interactions
	310	7.83 × 10^3^			−23.11	
	330	1.76 × 10^4^			−26.84	
**Fe_3_O_4_@Ag/TCS**	298	2.62 × 10^3^	25.61	149.78	−19.51	Hydrophobic interactions
	310	1.93 × 10^3^			−19.50	
	330	7.29 × 10^3^			−24.41	

## Data Availability

The data presented in this study are available on request from the corresponding author.
